# The Flavonoid Molecule Procyanidin Reduces Phase Separation in Model Membranes

**DOI:** 10.3390/membranes12100943

**Published:** 2022-09-27

**Authors:** Tsuyoshi Yoda

**Affiliations:** 1 Hachinohe Industrial Research Institute, Aomori Prefectural Industrial Technology Research Center, 1-4-43 Kita-inter-kogyodanchi, Hachinohe City 039-2245, Japan; tsuyohi_yoda@aomori-itc.or.jp; Tel.: +81-178-21-2100; 2 The United Graduate School of Agricultural Sciences, Iwate University, 3-18-8 Ueda, Morioka City 020-8550, Japan

**Keywords:** procyanidin, liposome, phase-separation, model biomembranes

## Abstract

Procyanidin extracted from fruits, such as apples, has been shown to improve lipid metabolization. Recently, studies have revealed that procyanidin interacts with lipid molecules in membranes to enhance lipid metabolism; however, direct evidence of the interaction between procyanidin and lipid membranes has not been demonstrated. In this study, the phase behaviors and changes in the membrane fluidity of cell-sized liposomes containing apple procyanidin, procyanidin B2 (PB2), were demonstrated for the first time. Phase separation in 1,2-Dioleoyl-sn-glycero-3-phosphocholine (DOPC)/1,2-dipalmitoyl-sn-glycero-3-phosphocholine (DPPC)/cholesterol ternary membranes significantly decreased after the addition of PB2. The prospect of applying procyanidin content measurements, using the results of this study, to commercial apple juice was also assessed. Specifically, the PB2 concentrations were 50%, 33%, and 0% for pure apple juice, 2-fold diluted apple juice, and pure water, respectively. The results of the actual juice were correlated with PB2 concentrations and phase-separated liposomes ratios, as well as with the results of experiments involving pure chemicals. In conclusion, the mechanism through which procyanidin improves lipid metabolism through the regulation of membrane fluidity was established.

## 1. Introduction

Understanding the interaction between procyanidins and the cell membrane requires the investigation of the receptors or channels stimulated in the gastrointestinal tract by procyanidin. Studies have reported that procyanidins interact with cell membranes to reduce oxidative stress [1] and increase membrane fluidity [2]. The release of procyanidins from liposomes has also been examined [3]. Procyanidins become part of the membrane when prepared with lipids to form liposomes. Liposomes contain a variety of lipids that mimic the lipid composition of cell membranes. They also contain a lipid bilayer structure like cell membranes, which is important as a cell membrane model. However, in previous studies, liposomes’ size was in the nanoscale range, which is relatively small [3]. Micrometer scale-based biological model membrane vesicles, known as cell-sized liposomes, have been used to examine the interaction between membranes and functional substances through direct observation under an optical microscope [4,5,6]. Cell-sized liposomes do not contain any protein or metabolic pathways. However, they are useful as a physicochemical model because they have a similar size to a cell and, thus the same curvature of the lipid bilayer structure. Based on the proposed model, functional proteins, such as receptors and channels, can move freely in the lipid bilayer. Over the past 20 years, the characterization of the structure of the lipid bilayer has progressed rapidly, with the raft structure considered to be one of the most popular proposed models [7,8]. Pike defined membrane rafts as small (10–200 nm), heterogeneous, highly dynamic, sterol- and sphingolipid-enriched domains that compartmentalize cellular processes. Small rafts can be stabilized to form larger platforms through protein–protein and protein–lipid interactions [9]. Clustering rafts may be observed on actual cultivated cells, and functions of raft domains were characterized to concern the signal transduction of cells [10,11]. Based on this model, functional proteins move into small compartments known as raft regions. The size of the actual raft is considered to be in the nanoscale range; however, in the case of cell signal enhancement, such as the activation of immunity rafts, clusters are formed that are micrometers in size. This phenomenon is observed in actual cells [10,11]. Although the definition of rafts is intended to apply specifically to microdomains in actual cells and not in model membranes, such as liposomes, which may be thought of differently, there are an overlapping set of rules [9]. Raft domains have been observed in micrometer-scale liposomes [12,13]. The formation of raft domains affects various lipids and sterol structures [14,15] and has been studied for active pathways, such as decreased domain stability by anesthetics. Simple or complex raft formation conditions have also been assessed, including optimized pH effects, as well as lipid types and charges [6,15,16].

Procyanidins are a type of polyphenolic substance that are components of functional foods. For example, theaflavins are highly indigestible compounds such as procyanidin that inhibit alfa glucosidase in the small intestine [17]. Food-derived polyphenols and similar functional compounds have been evaluated as potential targets for sugar and lipid absorption enhancement during digestion in humans [18]. Moreover, there are limited studies that address the inhibition by procyanidin. During lipid metabolism, procyanidin inhibits lipase activity. Procyanidin, from monomers to pentamers, exerts an inhibitory effect on lipase in vitro and in vivo. Such inhibitory effects are increased with increasing levels of polymerization [19]. Furthermore, in animal studies, triglyceride absorption was inhibited after a one-hour treatment with procyanidin [19]. However, the inhibitory mechanisms of procyanidin remain unknown, and varying degrees of polymerization may affect procyanidin activity differently [20]. In contrast, lipid accumulation in mice is prevented by a diet containing significant procyanidin levels, such as black soybeans and cacao fruits [20,21]. Previously reported mechanisms include the increased expression of PGC-1α and increased energy production that results in the inhibition of lipid accumulation [20,21]. Kamio et al. also found that a single dose of procyanidin increases energy metabolism by enhancing the expression of the *UCP-1* gene in brown adipose tissue, and neurotransmitters were involved in the mechanism of action [22]. Moreover, repeated administration of procyanidins promotes mitochondrial neoplasia with increased expression of the genes encoding PGC-1α and UCP-1 in skeletal muscle. Procyanidins may stimulate the sympathetic nervous system in the digestive tract, which significantly affects energy metabolism [23], causing the secretion of catecholamines and exerting a systemic metabolism-promoting effect [21]. Consequently, procyanidins may act primarily on receptors in the gastrointestinal tract and control metabolic regulation in peripheral tissues by altering signaling pathways [20]. Thus, the interaction between procyanidin and various receptors requires further delineation.

In this study, the phase behaviors and changes in the membrane fluidity of cell-sized liposomes containing apple procyanidin, procyanidin B2 (PB2), were demonstrated for the first time. 

## 2. Materials and Methods

### 2.1. Chemicals and Reagents

1,2-Dioleoyl-sn-glycero-3-phosphocholine (DOPC), 1,2-dipalmitoyl-sn-glycero-3-phosphocholine (DPPC), cholesterol (Chol), and dimethyl sulfoxide (DMSO) were purchased from the Tokyo Chemical Industry Co., Ltd. (Tokyo, Japan). Lissamine™ rhodamin B 1,2-dihexadecanoyl-sn-glycero-3-phosphoethanolamine and triethylammonium salt (Rhodamine DHPE) were obtained from Invitrogen. Ultrapure water, prepared by an RFD240NC purification system (ADVANTEC, Tokyo, Japan), was used for reagent preparation and glassware cleaning. Acetone was purchased from Wako Pure Chemical (Osaka, Japan). Chloroform was purchased from Kanto-chemical (Tokyo, Japan). PB2 was obtained from Fuji film Wako Pure Chemical (Osaka, Japan). Pure apple juice (1-L bottles) was purchased from the Farm Village Industry Federation of Aomori Prefectural Agricultural (Hirosaki, Japan).

### 2.2. Preparation of Liposomes Containing PB2

Acetone was used as a washing solution for the glass test tubes. Several different types of liposomes (giant unilamellar vesicles and model membranes/liposomes) were prepared. A slightly modified version of the method of natural swelling from dry lipid films was used, as described previously [24,25,26,27,28]. Mixtures of lipids, PB2, and Rhodamine DHPE were dissolved in chloroform in a glass test tube under argon gas to prepare a thin film. The glass test tubes were pre-washed with acetone and dried using a draft. They were then dried under vacuum for 3 h to produce thin lipid films. The films were then hydrated overnight with ultrapure water or PB2 solution at room temperature (20 °C). The PB2 solution was prepared according to a previously reported method [28] through dissolution in aqueous methanol [14.25% (*v*/*v*)]. A 1 mM stock solution was created and stored at −30 °C. When used in experiments, the methanol was diluted 5 times with ultrapure water. The final working solution had a concentration of 200 μM. The final concentration of the hydrated film was 200 μM for total lipid and PB2 and 5 μM Rhodamine DHPE. For PB2, instead of 10%–50% of lipids, the final concentration was 20–100 μM. The formation of unilamellar vesicles was highly dependent on the preparation conditions. The samples were carefully prepared, and the conditions were adjusted as described below. Thin lipid films were maintained in a vacuum before hydration with water. During hydration, the test tube was double wrapped with parafilm and aluminum foil to prevent oxidation and preserve fluorescence. The test tube was stored in a drawer at a constant temperature (at room temperature, 20 °C) in the dark until microscopic observation, which was performed within a week.

### 2.3. Microscopic Observations of Behaviors during Phase Separation of Liposomes

**Figure 1:**
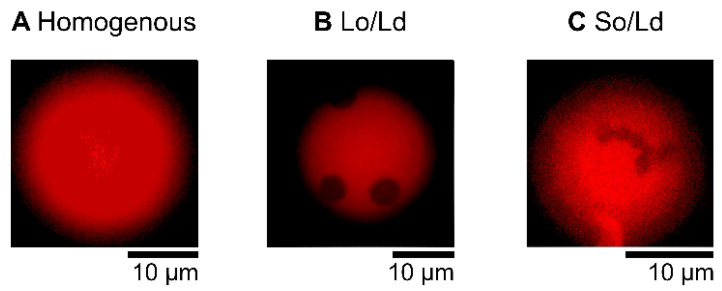
Microscopic images of multicomponent liposomes. Homogeneous (Ld) phase (DOPC/DPPC 50:50, (**A**), Lo/Ld phase separation (DOPC/DPPC/Chol 40:40:20, (**B**), and So/Ld phase separation (DOPC/DPPC 50:50, (**C**). Bright regions indicate the Ld phase (**A**–**C**), and dark regions indicate the So and Lo phases in (**B**,**C**), respectively. The Ld phase was labeled with a fluorescent dye (Rhodamine-DHPE). Rhodamine DHPE should be almost entirely distributed throughout the Ld phase [44].

The liposome solution (6 μL), prepared as described above, was placed in silicon wells (0.2 mm) on a glass slide and covered with a cover slip. Domain liposomes were observed using a fluorescence microscope (BX51, Olympus, Tokyo, Japan, [24] at room temperature. The microscope included an oil immersion objective lens (Uplan S-Apo, Olympus, Tokyo, Japan), LED excitation source (U-HGLGPS), and fluorescence mirror units with a dichroic mirror (410, 505, and 570 nm, respectively). The Ld phase was labeled with Rhodamine-DHPE (irradiated by green light, as shown in the red fluorescence region). Images of the phase-separated liposomes were obtained as pictures captured by the microscope camera (WRAYCAM-VEX830, Wraymer, Osaka, Japan). A minimum of 60 liposomes were observed for each type. Cell-sized liposomes (approximately 10 μm) were randomly selected. Liposomes were prepared at least three times, and we confirmed that there was no significant bias in each preparation. To observe the effects of PB2 on phase separation by fluorescence observation, PB2 was added to a DOPC/DPPC lipid mixture with a DOPC:DPPC ratio of 1:1, and to the DOPC/DPPC/Chol lipid mixture with a DOPC:DPPC:Chol ratio of 2:2:1. The resulting compositions were DOPC/DPPC/PB2 50:50:0, 45:45:10, 40:40:20, 35:35:30, 30:30:40, and 25:25:50 and DOPC/DPPC/Chol/PB2 40:40:20:0, 36:36:18:10, 32:32:16:20, 28:28:14:30, 24:24:12:40, and 20:20:10:50. These ratios were in accordance with a previous study [25]. The following three types of liposome states were identified from representative microscopic images by determining the presence and type of phase-separated domains: “homogenous,” “Liquid ordered (Lo)/Liquid disordered (Ld),” and “Solid ordered (So)/Ld” (Figure 1. In the Lo/Ld domain liposome, two types were observed. One contained circular Lo phase domains not dyed (black) in red with a fluorescent dye (Ld domain). The second was a circular domain dyed white with fluorescence and was observed in a Lo phase and not dyed (black) with fluorescence. The So/Ld domain exhibited anisotropic shapes surrounded by an Ld phase. Several studies have analyzed the phase state of membranes, and the equilibrium phase diagrams of ternary vesicles are well-characterized [25,26]. In an experiment used to investigate PB2 interactions for the phase separation of membranes, 6 μL of the liposome solution and 6 μL of 200 μM PB2 solution was poured into a test tube and gently mixed via soft tapping. Subsequently, 6 μL of the resultant mixture was used for microscopic observations [28].

### 2.4. Measurements of Membrane Fluidity in Liposome Membranes

The fluidity of membranes containing DOPC/DPPC/Chol and PB2 was measured using excitation Laurdan generalized polarization (GP) [25,26,29,30,31,32]. The Laurdan fluorescent label was used at 0.5% (molar ratio/ moral ratio). A Laurdan, 100 μM stock solution was prepared in chloroform at a final concentration of 1 μM. The final lipid concentration was 200 μM. The liposomes were observed at approximately 410 and 505 nm (Laurdan emission) using a dichroic mirror on the microscope (Olympus BX51 with a fluorescence unit attached, Olympus, Japan). The Laurdan GP value was defined as GP = (I420–460 nm − I510–550 nm)/(I420–460 nm + I510–550 nm), where I420–460 nm and I510–550 nm are the average fluorescence intensities of Laurdan detected at ranges of 420–460 and 510–550 nm, respectively, for >15 liposomes. The GP value was calculated by taking fluorescence images using Image J software [33], as our microscopic system could capture each fluorescence image taken by irradiation at two wavelengths. Each GP value was adjusted through the use of a specific correction factor for the experimental setup to measure the GP value of Laurdan in DMSO [30].

### 2.5. Estimated PB2 in Apple Juice and Its Application to Phase-Separated Liposomes

A 25 mL sample solution was prepared by mixing 5 mL of apple juice, 17.5 mL of acetone, and pure water [34,35,36]. Next, 20 μL of each sample solution was added to the DOPC/DPPC/Chol lipid mixture with a DOPC:DPPC:Chol ratio of 2:2:1. Each extracted solution and lipid solution were transferred to a glass test tube under argon gas to prepare a thin film. The glass test tubes were already washed with acetone and dried using a draft. They were then dried under vacuum for 3 h to produce thin lipid films. The films were then hydrated overnight with ultrapure water at room temperature (20 °C). The total lipid concentration was 0.2 mM, which was similar tp that of the experiment to simulate the 2.2 preparations of liposomes. The estimated PB2 content was approximately 0.2, 0.1, and 0 mM for pure apple juice, 2-fold diluted apple juice, and pure water, respectively, based on calculations from previous studies [34,35,36]. The PB2 concentration in the films was 50%, 33%, and 0% for pure apple juice, 2-fold diluted apple juice, and pure water, respectively. Microscopic observation methods were the same as described above.

### 2.6. Statistical Evaluation

Over 60 liposomes were observed for each composition, and the phase-separated structures were classified based on domain shape. The average values of triplicate experiments are shown in the figures. Each experiment was performed in triplicate. Given that 60 liposomes were sufficient to prevent accidental bias in the experiment, the differences were considered small enough to be ignored, and the error bars were not shown. GP values were measured as more than 100 pixels per liposome, and average GP (used over 15 liposomes) values and standard error were calculated. Graph preparation and statistical evaluation were performed using Microsoft Excel (Microsoft Office 2019).

## 3. Results and Discussion

This study aimed to reveal the mechanism underlying the activation of lipid metabolism by procyanidin. It was hypothesized that procyanidins change membrane fluidity and/or phase separation, leading to the activation of membrane receptors, which results in the activation of lipid metabolism. In this study, phase-separated cell-sized liposomes containing procyanidin were examined by microscopic observation. Procyanidin B2 (PB2) extracted from apples was used as a standard compound [34,35,36]. The liposomes were observed by microscopy, and phase separation with respect to various PB2 concentrations was determined. Laurdan was used previously to measure the fluidity of different lipid phases [29]. Thus, the membrane fluidity of cell-sized liposomes with different concentrations of PB2 was evaluated using the fluorescence probe Laurdan [24,25,29,30,31,32]. The results revealed a variation in the phase separation and membrane fluidity induced by procyanidin as well as by related membrane receptor activation. Our findings provide insights into the mechanisms underlying the function of procyanidin, including the activation of lipid metabolism.

This study focused on phase-separated domain structures in the membranes of liposomes by fluorescence observation. The classification of each phase-separated state, using morphological detection, is described in the Materials and Methods Section. The percentage of phase-separated structures was confirmed visually. We expected to observe the same phase behavior for the same liposome composition, which would reflect ideal conditions. Our liposome preparation used natural swelling methods, and although lipid mixture preparation was carefully performed, some variation may occur in each liposome. Nonetheless, phase-separation tendencies obeyed the lipid mixture condition, as most phase-separation research in liposomes reported [25,26,37,38]. Previously, variations in the physicochemical properties of liposomes were found even in the same preparation [27]. Dispersion is likely the result of a wide variation in the mixed lipid fraction during liposome preparation.

**Figure 2:**
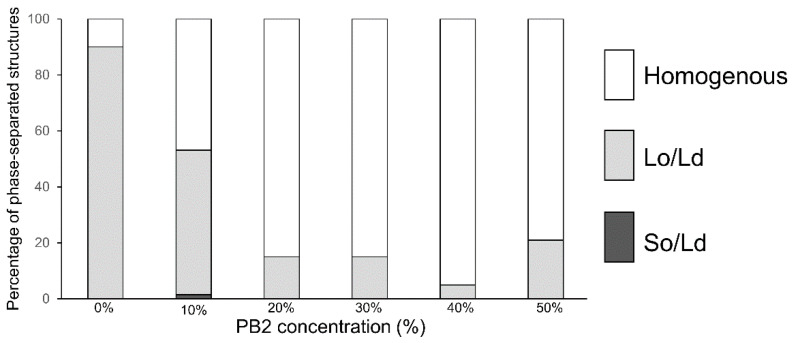
The percentage of phase-separated structures. 1,2-Dioleoyl-sn-glycero-3-phosphocholine (DOPC)/1,2-dipalmitoyl-sn-glycero-3-phosphocholine (DPPC)/cholesterol (Chol) with various concentrations of pro-cyanidin b2 (PB2) are shown. White, bright gray, and dark gray bars denote homogenous, liquid-ordered (Lo)/liquid-disordered (Ld), and solid-ordered (So)/Ld phases, respectively. Lipid compositions of the liposomes for each observation were DOPC/DPPC/Chol/PB2 40:40:20:0, 36:36:18:10, 32:32:16:20, 28:28:14:30, 24:24:12:40, and 20:20:10:50. According to previous studies, DOPC/DPPC 40:40:20 was used as a control for Lo/Ld phase-separation [12,13,25,26].

In the control experiment, using 1,2-Dioleoyl-sn-glycero-3-phosphocholine (DOPC)/1,2-dipalmitoyl-sn-glycero-3-phosphocholine (DPPC)/Cholestrol (Chol) without PB2 liposomes, 90% of the Lo/Ld phase-separated liposomes (Figure 1) were initially observed. The results were consistent with that of previous studies [12,13,25,26], confirming the efficiency of our observations. Although more than half of the liposomes exhibited phase-separated structures, the ratio of Lo/Ld phase-separated domains was decreased, whereas that of the homogeneous phase (Figure 1 and Figure 2) was increased by adding 10% PB2. Based on the percentages of phase-separated structures (sum of So/Ld and Lo/Ld), which were 15% (PB2 20%), 15% (PB2 30%), 5% (PB2 40%), and 21% (PB2 50%), the presence of PB2 destabilized the Lo/Ld phase-separated structures and enhanced the mixture of the lipids. Liposomes containing 50% PB2 exhibited a slightly higher ratio of phase-separated structures [25]. Previous studies have reported the use of nano-scaled liposomes with procyanidin at limited concentrations of 20 mol% to 30 mol% [1,2]. Furthermore, liposomes have been prepared with other compounds, such as cholesterol of up to 50% [39,40,41]. Therefore, although 50 mol% is a relatively high concentration compared with previous studies of liposomes containing procyanidin, the concentration is sufficient to generate liposomes. Increased PB2 reached 50%, suggesting that PB2 may quench fluorescence. Previously, we observed a similar pattern with membranes containing theaflavin, which has a similar structure to PB2 [28]. These phenomena are consistent with previous studies that showed that PB2 quenches fluoresce intensity [42,43].

**Figure 3:**
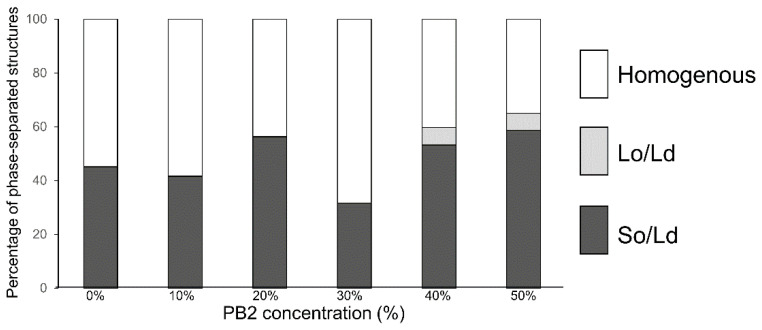
The percentage of phase-separated structures. 1,2-Dioleoyl-sn-glycero-3-phosphocholine (DOPC)/1,2-dipalmitoyl-sn-glycero-3-phosphocholine (DPPC) with various concentrations of procyanidin b2 (PB2) are shown. White, bright gray, and dark gray bars denote homogenous, liquid-ordered (Lo)/liquid-disordered (Ld), and solid-ordered (So)/Ld phases, respectively. Lipid compositions of the observed liposomes in the experiment were DOPC/DPPC/PB2 50:50:0, 45:45:10, 40:40:20, 35:35:30, 30:30:40, and 25:25:50. Based on previous studies, So/Ld phase separation liposomes, DOPC/DPPC 50:50 were used as a control [12,25,26].

In the control experiment for DOPC/DPPC without PB2 liposomes, 45% of So/Ld phase-separated liposomes (Figure 3) were observed. This ratio was slightly lower compared with the phase-separated liposomes in previous studies [25], although phase-separated liposomes were definitely observed. The percentage of So/Ld fluctuated with increasing PB2 concentrations (Figure 3). PB2 exerts certain So/Ld phase-separation effects on membranes, although these effects are not dependent upon PB2 concentration. Although this is an interesting phenomenon, the underlying mechanisms remain unclear. A liquid-ordered phase was observed at 40% and 50% PB2. This was considered significant, as PB2 negatively affects the stability of the liquid-ordered phase, whereas it did not significantly affect the solid-ordered phase. Another important finding was that a relatively higher PB2 concentration induced a liquid-ordered phase on the membrane in the absence of Chol (Figure 3).

**Figure 4:**
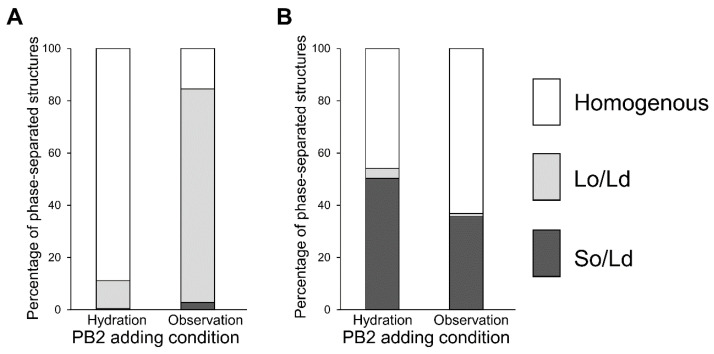
Percentage of phase-separated structures. 1,2-Dioleoyl-sn-glycero-3-phosphocholine (DOPC)/1,2-dipalmitoyl-sn-glycero-3-phosphocholine (DPPC)/cholesterol (Chol) with various additions of procyanidin b2 (PB2) are shown. Lipid compositions of the observed liposomes in the experiment were DOPC/DPPC/Chol (**A**) 40:40:20 and (**B**) 50:50:0.

Next, we investigated whether procyanidin affects membranes owing to the escape from the membrane or insertion into the membrane. The procyanidin solution was used during liposome hydration and liposome observation, as shown in Figure 4. These experiments allowed us to determine whether procyanidin binds to membranes during hydration or observation. Procyanidin added under hydration conditions led to 50% procyanidin in the membranes, whereas procyanidin added during observation led to a liposome without procyanidin. Because hydration occurs over hours, the water facilitates the binding of procyanidin to the membranes. In contrast, observation occurs over minutes; thus, the procyanidin does not bind to the membranes in this short period. 

**Figure 5:**
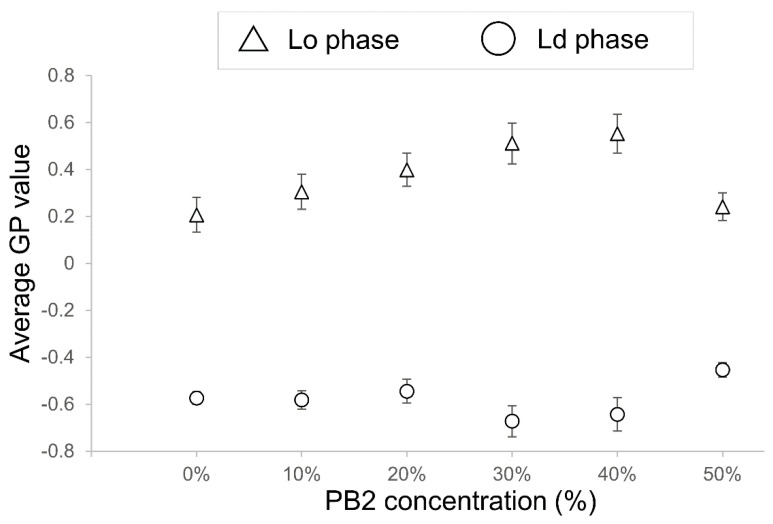
Average GP values of procyanidin b2-containing membranes (n = 15). High GP value means low fluidity of the membranes, whereas low GP values mean a high fluidic state in the membrane. Triangles represent GP values of liquid-ordered (Lo) phase membranes, and circles indicate GP values of liquid-disordered (Ld) phase membranes. The bar on each marker shows the standard error of the GP values for each component. Lipid compositions for the liposomes were DOPC/DPPC/Chol/PB2 40:40:20:0, 36:36:18:10, 32:32:16:20, 28:28:14:30, 24:24:12:40, and 20:20:10:50. Based on previous studies, DOPC/DPPC/20 40:40:20 liposomes were used as a control for Lo/Ld phase separation liposomes [12,25,26].

The effect of PB2 on the membrane fluidity of liposomes was examined next by determining the GP values. A high GP value indicates low fluidity of the membranes, whereas low GP values indicate a high fluidic state in the membranes. The GP values are summarized in Figure 5. For PB2 concentrations of 10%, 20%, and 50%, the values were not significantly different; however, when the PB2 concentration was set to 30% and 40%, the values were significantly different. As the value of the Lo phase increased, and that of the Ld phase decreased significantly, a fluidity change occurred in the Lo phase and Ld phase at 20% and 30% PB2, respectively. Although phase-separated liposomes decreased dramatically when PB2 was increased from 10% to 20% (Figure 2), GP decreased significantly when PB2 increased from 20% to 30%. The difference in liquid-ordered and disordered phase fluidities appears to be an important factor for phase separation on the membranes [25,45]. These results are in accordance with previous studies showing that PB2 concentrations of approximately 10% to 30% affect membrane fluidity and result in phase separation. The GP value of Lo at 50% PB2 was very similar to that at zero and for a low percentage of PB2. Thus, it appears that a higher PB2 concentration causes increased fluidity and disturbance to the forming of a liquid-ordered phase.

The results regarding GP variations at increased PB2 concentrations in the Ld phase were in agreement with the results of lipid bilayer vesicles formed from 1,2-Dimyristoyl-sn-glycero-3-phosphocholine, which were approximately 100 nm in size and contained procyanidin trimers (PB3) [46]. In that study, although PB3 was in the molar concentration range, corresponding to approximately 10% to 20% lipid, this concentration range could be considered comparable to the concentration range observed in the present study because trimers were used instead of dimers.

Based on the observed decrease in the ratio of Lo/Ld phase-separated liposomes and the fluidity of the PB2 containing liposomes, the mechanism for the stability of So/Ld phase-separated liposomes may not be dependent upon membrane fluidity. Sugahara et al. reported similar results for the phase separation of cell-sized liposomes containing local anesthetics (LAs) [25]. They found that LAs destabilized the Lo/Ld phase separation but did not affect So/Ld phase separation. They hypothesized that LAs may change membrane fluidity for So/Ld phase-separated membranes. Although LAs increased the fluidity of the So phase and decreased the Ld phase, phase separation was unchanged because of the difference in fluidity between So and Ld was large. The same mechanisms should be at play on PB2-containing membranes in the present study; however, we observed Lo/Ld phase separation in membranes containing DOPC/DPPC/PB2 without Chol when the PB2 concentration was increased (Figure 3). Because the present study resulted in interesting findings, further elucidation of liposome phase-separated mechanisms containing procyanidin will be pursued in the future.

In this study, we discovered that procyanidin induced a change in membrane fluidity and a decrease in Lo/Ld phase separation at selected concentrations. Procyanidin may modify lipid metabolism through the transformation of receptors or/and through their channel-related activities. A previous study reported that procyanidin inhibited the regulation of potassium ions through ion channels [47]. Other studies indicated that the function of ion channels for potassium is influenced by the lipid phase and lipid transition [48,49,50]. Thus, it seems reasonable that PB2 regulates ion channels through a lipid phase transition and lipid phase separation. These studies reported changes in lipid phase separation on membranes and channel activity. Therefore, at least one mechanism for ion channel activation may involve lipid phase transition and lipid phase separation. It was also reported that lipid phase separation is influenced by receptor activity. A recent study found that receptor activity deceased through perturbation of the raft structure by procyanidin [51,52]. Other studies support another kind of receptor activity affected by lipid phase separation [53,54]. In the present study, we demonstrated that procyanidin did not significantly alter So/Ld phase separation; however, some studies have concluded that Lo/Ld and So/Ld phase separation exhibit different properties [55,56].

**Figure 6:**
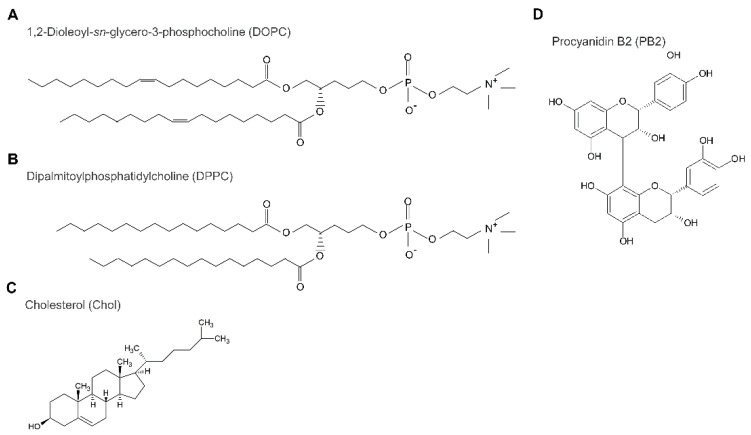
Structure of lipids, cholesterol, and procyanidin b2; 1,2-Dioleoyl-sn-glycero-3-phosphocholine (DOPC; **A**), 1,2-dipalmitoyl-sn-glycero-3-phosphocholine (DPPC; **B**), cholesterol (Chol; **C**), and procyanidin B2 (PB2; **D**).

By analyzing data from previous studies on lipid phase separation in the presence of alien molecules in the bilayer, with these data obtained experimentally and via computational simulations, it is possible to explain the influence of procyanidin on lipid phase separation at the molecular level as follows. Based on previous studies [52,55,56], it has been hypothesized that procyanidin can decrease Lo/Ld phase separation rather than So/Ld phase separation. Procyanidin affects Lo/Ld membranes but not So/Ld membranes. As procyanidin contains a carbon ring and a hydroxy group, it appears to have a higher affinity for Lo domains (Figure 6). The position of procyanidin should be on the interface of the hydrophilic and hydrophobic regions in lipids on the Lo domain of the membranes [52]. The results of the study revealed that the thickness of the bilayer membrane was approximately 6.0 and 3.0 nm from the center to the water end of the membrane in a layer. Procyanidin was attached approximately 2.0 to 2.5 nm from the center part of the membranes and was positioned at the cholesterol oxygen atom between the phosphorus atom of the phospholipid. In this position, procyanidin could interact with membrane lipids in the Lo domain, although a small amount of procyanidin may not render Lo domain formation unstable. The study indicated that the procyanidin molecule attracted a membrane lipid, but the galloyl group bound to procyanidin was inserted into the Lo domain of the membranes. In the present study, we demonstrated that a small concentration of procyanidin has a substantial effect on domain reduction, which is smaller compared with a high concentration of procyanidin (Figure 2). We counted 180 liposomes for each experiment, which included 60 liposomes at least thrice. We hypothesized that 50% PB2 quenched fluorescence intensity because, at relatively higher concentrations, PB2 aggregation may cause slightly higher phase separation. Although 40% PB2 is a relatively lower concentration than 50% PB2, the aggregation of PB2 might not occur in membranes often; thus, phase separation would seldom be observed. The results of the present study are in agreement with those of a previous study [52].

The mechanism for the phase behavior concerning how the presence of procyanidin could alter fluidity change-based membrane phase properties may be explained as follows. The fluidity of the Lo domain is higher compared with that of the So domain, and procyanidin may be incorporated in the Lo domain. The Lo domain is rich in DPPC and Chol, and when procyanidin is inserted, the fluidity is increased. As the fluidity of the Lo domain increases, the difference in fluidity between the Lo and the Ld domains (DOPC rich domain) will be small, resulting in an increased homogenous liposome ratio. In So/Ld phase-separated liposomes, the So domains are rich in DPPC with a saturated carbon chain and solid-like state. Although procyanidin contains a carbon ring and hydroxy group, it does not commonly incorporate into the So domain. Procyanidin can incorporate into the Ld domain containing a DOPC-rich domain in So/Ld phase-separated liposomes. The fluidity difference between the So and Ld domains were not changed significantly, and the So/Ld phase-separated liposome ratio was not modified significantly. At relatively higher concentrations of procyanidin, some procyanidin incorporates into the DPPC-rich region. A small ratio of procyanidin behaves like Chol when preparing a Lo domain with DPPC, and a small ratio of Lo/Ld phase-separated liposomes was observed (Figure 4). A previous study reported that Lo/Ld phase separation was induced by photoisomerization of lipids [57]. This study indicated that isomerized photo-responsive lipids could stabilize to form phase-separated domains. A solid domain modification to the Lo/Ld domain resulted in a decrease in lipid packing, as an inverse phenomenon of Lo/Ld to So/Ld phase separation was reported to result in increased lipid packing by osmotic pressure [37]. When PB2 causes decreased lipid packing in membranes, it was observed that the Lo/Ld phase-separation ratio was decreased (Figure 2). Indeed, a relevant phenomenon has been previously reported. Muraoka et al. reported that changes in membrane pressure could induce the functional activation of synthesized transmembrane multi-block amphiphiles as ion channels using cell-sized liposomes prepared from DOPC with these amphiphiles [58]. Recently, Wang et al. also reported that PB2 and related compounds downregulated the activity of enzymes related to lipid metabolism [52]. This process was caused by the reduced expression of genes encoding such enzymes and resulted in perturbation of the lipid raft by interactions between procyanidin derivatives and lipid molecules to cause reduced receptor activation [52]. Wang et al. utilized cells with metabolic activities as well as computer simulations. Importantly, the findings of the present study are in accordance with the findings of Wang et al., although model membrane systems without metabolic activity were used in the current study [51]. Kamio et al. discovered that increased procyanidin enhances the expression of the gene encoding UCP-1 [22]. As UCP-1 is localized within mitochondrial membranes, procyanidin may synergistically enhance the activation of UCP-1 to induce an alteration in membrane properties to optimize the relevant conditions, such as increased membrane fluidity and less packing.

**Figure 7:**
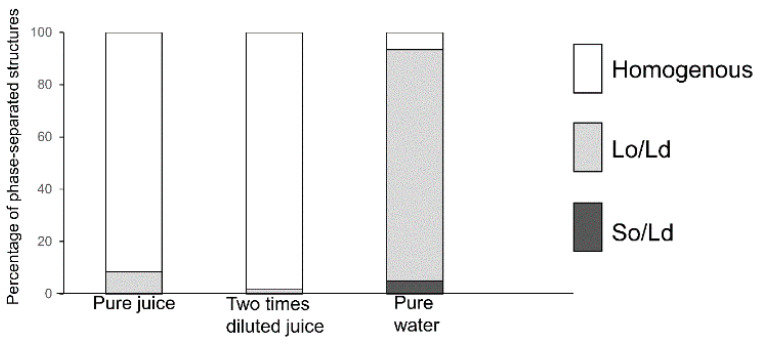
The percentage of phase-separated structures; 1,2-Dioleoyl-sn-glycero-3-phosphocholine (DOPC)/1,2-dipalmitoyl-sn-glycero-3-phosphocholine (DPPC)/cholesterol (Chol) with several concentrations of procyanidin b2 (PB2) containing pure juice, two times diluted juice, and pure water are represented. White, bright gray, and dark gray bars denote homogenous, Lo/Ld, and So/Ld phase, respectively.

The results of Lo-Ld phase-separated liposome reduction (Figure 2) may be applied for the detection of PB2 or procyanidin as a ratio of the lipids that are required to prepare liposomes. Apples are the second most produced fruit in the world, followed by bananas [59]. Apples are rich in procyanidins, and the production volume of concentrated juice is the highest among juice varieties [60]. For this reason, we considered apple juice as the target from the perspective of the demand for procyanidin content measurement and quality maintenance. Previous studies from our institute revealed that to make concentrated apple juice, freezing and melting must be performed to obtain a high concentration of procyanidin. The effectiveness and container shape change increased the concentration ratio [36,61]. In these studies, the concentration of procyanidin was monitored using high-performance liquid chromatography, which is not a cost- or time-effective approach. Recently, apple procyanidin was monitored using Raman spectroscopy and multivariate calibration analysis [62]. Although this study detected procyanidin with non-destructed apple, procyanidin in apple juice was not measured. Compared with these research developments and quality assurance procedures, the approach suggested in the current study may be a candidate as a faster and cost-effective detection method, as it enables adequate mixing of lipids to prepare and observe liposomes. Extracted PB2 was then used in apple juice and applied to phase-separated liposomes before observation. The summarized results are presented in Figure 7. Pure apple juice, 2-fold diluted juice, and pure water (used as a negative control) were evaluated. The PB2 concentrations were 50%, 33%, and 0% for pure apple juice, 2-fold diluted apple juice, and pure water, respectively. The correlation of PB2 concentrations and of phase-separated liposome ratios are in accordance with Figure 2. As only three conditions were evaluated, the data are considered preliminary. Nevertheless, the approaches using phase-separated liposomes may be applied to the measurement of procyanidin. This study had some limitations. Although procyanidin was not measured in this study, the procyanidin content in our sample was similar to that found in apple juice [36]. Future research should include comparisons of the estimated data with a rigorous determination of calibrated methods. In addition, it will be necessary to estimate the purity of procyanidin and extracted impurities as well as to investigate the impact of extracted impurities on membrane phase separation.

## 4. Conclusions

We discovered that Laurdan generalized polarization was dependent upon procyanidin B2 concentration as an indicator of membrane fluidity. Based on our observation of the decrease in liquid-ordered/liquid-disordered phase separation and membrane fluidity in procyanidin B2-containing liposomes, solid ordered/liquid disordered-phase separation does not depend on procyanidin B2 concentration. The present study revealed that procyanidin induced fluidic properties on the membrane. Based on the results, procyanidin may transform receptors or their channel-related activities to improve lipid metabolism. Previous studies support the activities of both receptors and ion channels affected by the lipid phase in the membrane [48,49,50,53,54]. A correlation between PB2 concentrations and phase-separated liposome ratios was observed when using actual juice. This finding could lead to the development of a rapid measurement tool for procyanidin concentrations. Our findings not only enhance our understanding of the functional mechanism of procyanidin through the characterization of the biophysical aspects of lipid membranes but also suggest a rapid and cost-effective approach for the measurement of procyanidin.

## Data Availability

The research data have been provided in the manuscript.
